# 

**Published:** 1903-03

**Authors:** 


### BOOKS RECEIVED.

Transactions of the American Gynecological Society. Twenty-seventh annual meeting held at Alantic City, May 27–29, 1902. J. Riddle Goffe, M. D., Secretary. Philadelphia: William J. Dornan, Printer. 1902.

Lea's Series of Medical Epitomes A Manual of Obstetrics for Students and Practitioners. By W. P. Manton, M. D., Adjunct-Professor of Obstetrics and Professor of Clinical Gynecology, Detroit College of Medicine. In one 12mo volume of 265 pages, with 82 illustrations. Philadelphia and New York: Lea Brothers & Co. 1903. [Cloth, $1.00.]

A Text-Book of Practical Medicine. For the Use of Students and Practitioners. By William Gilman Thompson, M. D., Professor of Medicine in Cornell University Medical College, Physician to the Presbyterian Hospital, Bellevue Hospital, New York. New (second) edition, thoroughly revised. In one 8vo volume of 1,104 pages, with 62 illustrations. Philadelphia and New York: Lea Brothers & Co. 1903. [Cloth, $5.00; leather, $6.00; half morocco, $6.50 net.]

The American Year-Book of Medicine and Surgery for 1903. A Yearly Digest of Scientific Progress and Authoritative Opinions in all Branches of Medicine and Surgery, drawn from journals, monographs and textbooks of the leading American and foreign authors and investigators. Arranged, with critical editorial comments, by eminent American specialists, under the editorial charge of George M. Gould, A. M., M. D. In two volumes—Volume I., including General Medicine; 8vo, 700 pages, fully illustrated. Volume II., General Surgery; 8vo, 670 pages, fully illustrated. Philadelphia, New York, London: W. B. Saunders & Co. 1903. [Per volume: cloth, $3 00 net; half morocco, $3.75 net.]

Blakiston's Quiz Compends. A Compend of Diseases of Children. Especially adapted for the use of Medical Students. By Marcus P. Hatfield, A. M., M. D., Emeritus Professor of Diseases of Children, Northwestern University Medical School; Physician to Wesley Hospital, Home for Crippled Children, Chicago Orphan Asylum, Chicago. Duodecimo, pp. 241. Third edition, thoroughly revised, with colored plate. Philadelphia: P. Blakiston's Son & Co. 1903. [Price, 80 cents.]

Cushny's Pharmacology and Therapeutics. A Textbook of Pharmacology and Therapeutics; or the Action of Drugs in Health and Disease. By Arthur R. Cushny, A. M., M. D, Professor of Materia Medica and Therapeutics, University of Michigan, Department of Medicine and Surgery, Ann Arbor, Mich. Third edition, revised and enlarged. In one 8vo volume of 750 pages, with 52 engravings. Philadelphia and New York: Lea Brothers & Co. 1903. [Cloth, $3.75 net; leather, $4.75 net.]

Lea's Series of Medical Epitomes. An Epitome of Physiology. For Students and Practitioners of Medicine. By Theodore C. Guenther, M. D., Assistant Physician, Norwegian Hospital, Brooklyn, and Augustus E. Guenther, B. S., formerly Assistant in Physiology in the Medical Department, University of Michigan, Ann

Arbor. Series edited by Victor C. Pedersen, A. M., M. D , Clinical Assistant in Surgery at the New York Polyclinic Medical School and Hospital. In one 12mo volume of 250 pages, with 57 engravings. Philadelphia and New York: Lea Brothers & Co. 1903. [Cloth, $1.00 net.]

International Clinics. A quarterly of Illustrated Clinical Lectures and especially prepared Articles on Medicine, Neurology, Surgery, Therapeutics, Obstetrics, Pediatrics, Pathology, Dermatology, Diseases of the Eye, Ear, Nose, and Throat, and other Topics of Interest to Students and Practitioners, by leading members of the medical profession throughout the world Edited by Henry W. Cattell, A.M., M.D., Philadelphia. With the collaboration of John B. Murphy, M. D., Chicago; Alexander D. Blackader, M. D., Montreal; H. C. Wood, M. D., Philadelphia; T. M. Rotch, M. D., Boston; E. Landolt, M. D., Paris; Thomas G. Morton, M. D., Philadelphia; James J. Walsh, M. D., New York; J. W. Ballantyne, M. D., Edinburgh, and John Harold, M. D., London. With regular correspondents in Montreal, London, Paris, Leipsic and Vienna. Volume IV. Twelfth series. Philadelphia and London: J. B. Lippincott Co. [Cloth, $2.00.]

Huntington's Abdominal Anatomy. The Anatomy of the Human Peritoneum and Abdominal Cavity considered from the standpoint of Development and Comparative Anatomy. By George S. Huntington, M. D., Professor of Anatomy in the College of Physicians and Surgeons, Columbia University, New York City. In one handsome quarto volume of 590 pages, including 300 full-page plates in colors and monochrome, containing 582 figures. Philadelphia and New York: Lea Brothers & Co. 1903. [De luxe edition, gilt top, uncut edges, $10.00 net.]

Clinical Treatises on the Pathology and Therapy of Disorders of Metabolism and Nutrition. By Prof. Dr. Carl von Noorden, Senior- Physician to the City Hospital in Frankfort a. M. Authorised American edition translated under the direction of Boardman Reed, M.D , Professor of Diseases of the Gastrointestinal Tract, Hygiene and Climatology, Department of Medicine, Temple College, Philadelphia. Part I. Obesity. The Indications for Reduction Cures. Duodecimo, pp. 59. New York: E B. Treat & Company. 1903. [Price, 50 cents.]

Clinical Treatises on the Pathology and Therapy of Disorders of Metabolism and Nutrition. By Prof. Dr. Carl von Noorden, Physician-in-chief to the City Hospital, Frankfort a. M. Authorised American edition translated under the direction of Boardman Reed, M.D., Professor of Diseases of the Gastrointestinal Tract, Hygiene and Climatology, Department of Medicine, Temple College; Physician to the Samaritan Hospital, Philadelphia. Part II., Nephritis. Duodecimo, pp. 112. New York: E. B. Treat & Company. 1903. [Price, $1.00.]

Diseases of the Skin. Their Description, Pathology, Diagnosis, and Treatment. With special reference to the Skin Eruptions of Children and an Analysis of 15,000 cases of Skin Disease. By H. Radcliffe-Crocker, M. D , (Lond.), F. R. C. P., Physician for Diseases of the Skin in University College Hospital; Honorary Member of the American Dermatological Society; Examiner in Medicine, Apothecaries' Hall, London. Third edition, revised and enlarged. With four plates and 112 illustrations. Octavo, pp. 1,466. Philadelphia: P. Blakiston's Son & Co. 1903. [Price, $5.00.]

Obstetrics. A Textbook for the use of Students and Practitioners By J. Whitridge Williams, Professor of Obstetrics, Johns Hopkins University; Obstetrician-in-Chief to the Johns Hopkins Hospital; Gynecologist to the Union Protestant Infirmary, Baltimore. Octavo, pp. 867. With eight colored plates and 630 illustrations in the text New York and London: D. Appleton & Company. 1903. [Price, $6.00.]

